# Early versus delayed coronary angiography in patients with out-of-hospital cardiac arrest and no ST-segment elevation: a systematic review and meta-analysis of randomized controlled trials

**DOI:** 10.1007/s00392-023-02264-7

**Published:** 2023-07-27

**Authors:** Fardin Hamidi, Elaaha Anwari, Christian Spaulding, Caroline Hauw-Berlemont, Aurélie Vilfaillot, Ana Viana-Tejedor, Karl B. Kern, Chiu-Hsieh Hsu, Brian A. Bergmark, Arman Qamar, Deepak L. Bhatt, Remo H. M. Furtado, Peder L. Myhre, Christian Hengstenberg, Irene M. Lang, Norbert Frey, Anne Freund, Steffen Desch, Holger Thiele, Michael R. Preusch, Thomas A. Zelniker

**Affiliations:** 1 Division of Cardiology, Medical University of Vienna, Währinger Gürtel 18-20, 1090 Vienna, Austria; 2 Department of Cardiology, European Hospital Georges Pompidou, Assistance Publique–Hôpitaux de Paris, Paris Cité University, Sudden Cardiac Death Expert Center, Paris, France; 3 Medical Intensive Care Unit, European Hospital Georges Pompidou, Assistance Publique–Hôpitaux de Paris, Université Paris Cité, Paris, France; 4 Biostatistique et Santé Publique, European Hospital Georges Pompidou, Assistance Publique–Hôpitaux de Paris, Paris, France; 5 Acute Cardiac Care Unit, Department of Cardiology, University Hospital Clínico San Carlos, Madrid, Spain; 6 University of Arizona Sarver Heart Center, Tucson, AZ USA; 7 TIMI Study Group, Cardiovascular Division, Brigham and Women’s Hospital and Harvard Medical School, Boston, USA; 8 Cardiovascular Outcomes Research and Innovation Laboratory, Section of Interventional Cardiology and Vascular Medicine, NorthShore University Health System, Evanston, USA; 9 Mount Sinai Heart, Icahn School of Medicine at Mount Sinai Health System, New York, NY USA; 10 Brazilian Clinical Research Institute, Sao Paulo, Brazil; 11 Instituto do Coracao (InCor), Hospital das Clinicas da Faculdade de Medicina, Universidade de Sao Paulo, Sao Paulo, Brazil; 12 Department of Medicine, Division of Cardiology, Akershus University Hospital and K.G. Jebsen Center for Cardiac Biomarkers, University of Oslo, Oslo, Norway; 13 Department of Cardiology, Angiology, and Pneumology, University Hospital of Heidelberg, Im Neuenheimer Feld 410, 69120 Heidelberg, Germany; 14 DZHK (German Centre for Cardiovascular Research), Partner Site Heidelberg/Mannheim, Heidelberg, Germany; 15 Heart Center Leipzig at University of Leipzig and Leipzig Heart Institute, Leipzig, Germany

**Keywords:** Out-of-hospital cardiac arrest, Coronary angiography, Critical care medicine, Percutaneous coronary intervention

## Abstract

**Background:**

Recent randomized controlled trials did not show benefit of early/immediate coronary angiography (CAG) over a delayed/selective strategy in patients with out-of-hospital cardiac arrest (OHCA) and no ST-segment elevation. However, whether selected subgroups, specifically those with a high pretest probability of coronary artery disease may benefit from early CAG remains unclear.

**Methods:**

We included all randomized controlled trials that compared a strategy of early/immediate versus delayed/selective CAG in OHCA patients and no ST elevation and had a follow-up of at least 30 days. The primary outcome of interest was all-cause death. Odds ratios (OR) were calculated and pooled across trials. Interaction testing was used to assess for heterogeneity of treatment effects.

**Results:**

In total, 1512 patients (67 years, 26% female, 23% prior myocardial infarction) were included from 5 randomized controlled trials. Early/immediate versus delayed/selective CAG was not associated with a statistically significant difference in odds of death (OR 1.12, 95%-CI 0.91–1.38), with similar findings for the composite outcome of all-cause death or neurological deficit (OR 1.10, 95%-CI 0.89–1.36). There was no effect modification for death by age, presence of a shockable initial cardiac rhythm, history of coronary artery disease, presence of an ischemic event as the presumed cause of arrest, or time to return of spontaneous circulation (all P-interaction > 0.10). However, early/immediate CAG tended to be associated with higher odds of death in women (OR 1.52, 95%-CI 1.00–2.31, P = 0.050) than in men (OR 1.04, 95%-CI 0.82–1.33, P = 0.74; P-interaction 0.097).

**Conclusion:**

In OHCA patients without ST-segment elevation, a strategy of early/immediate versus delayed/selective CAG did not reduce all-cause mortality across major subgroups. However, women tended to have higher odds of death with early CAG.

**Graphical abstract:**

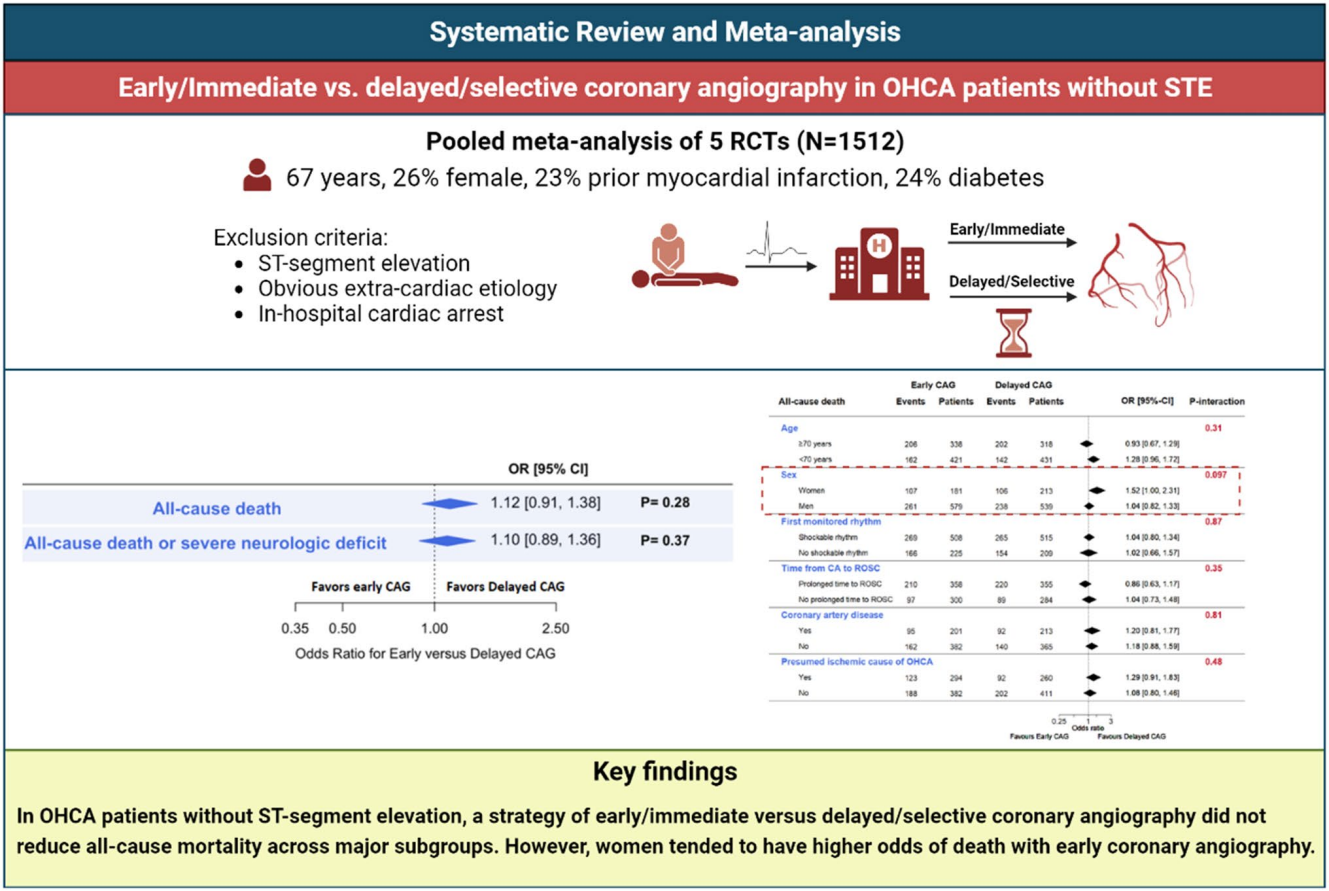

**Supplementary Information:**

The online version contains supplementary material available at 10.1007/s00392-023-02264-7.

## Background

Despite advances in resuscitation and critical care management, mortality rates in patients with out-of-hospital cardiac arrest (OHCA) remain high [1]. Although coronary artery disease and myocardial infarction are the most prevalent causes of OHCA, patients with OHCA and no ST-segment elevation constitute a heterogeneous group, making it challenging for clinicians to determine the appropriate indication and optimal timing of coronary angiography [2–4]

Although early/immediate coronary revascularization may improve myocardial function and prevent cardiogenic shock or life-threatening arrhythmia in patients with an acute coronary syndrome [5], recent randomized controlled trials did not show benefit of early/immediate coronary angiography over a delayed/selective strategy in an OHCA patient population without ST-segment elevation [1, 6–10]. However, it remains unclear whether some patients, such as those with a high pre-test probability of coronary artery disease, may benefit from an early or immediate strategy of coronary angiography [11]. Also, there is growing evidence of sex-based differences and disparities in patient characteristics, risk factors, management, and outcomes between men and women with OHCA. Women with OHCA are more likely to present with a non-shockable rhythm, to be older, have more comorbidities, are less likely to be resuscitated by bystanders, receive coronary angiography and targeted temperature management, and have poorer outcomes than men [11–16].

Because no single trial has been powered to test for effect modification across subgroups, we aimed to pool data from all randomized controlled trials that compared an early/immediate versus a delayed/selective strategy of coronary angiography in OHCA patients without ST-segment elevation to obtain more reliable estimates overall and in relevant subgroups.

## Materials and methods

### Search strategy

The present meta-analysis was done using the methods proposed in the Preferred Reporting Items for Systematic Reviews and Meta-Analysis statement (PRISMA-P) [17]. We performed a comprehensive data search of randomized-controlled trials that compared early/immediate versus delayed/selective coronary angiography with a follow-up time of at least 30 days in patients with OHCA and no ST-segment elevation from inception to 16.07.2022 on PubMed and EMBASE. Data search and extraction were conducted by 2 independent reviewers (F. H., E. A.) using a standardized data form, and any discrepancies were resolved by consensus or consulting a third reviewer (T. A. Z.). When endpoints (including those for subgroups) were not reported, the respective trial authors were contacted. The search algorithm is presented in detail in the appendix. Risk of bias was assessed using the Cochrane tool of risk of bias assessment Version 2 [18].

### Outcomes

The primary outcome of interest was all-cause mortality. The secondary outcome of interest was the composite of all-cause death or neurological deficit (defined as a cerebral performance category score of more than 2) (Supplemental Table 1).

### Statistical analysis

Pooled odds ratios (OR) and the corresponding 95% confidence intervals (CI) were calculated for the primary and secondary outcomes using a fixed effects model. In addition, sensitivity analyses were performed using random effect models by utilizing the method of residual maximum likelihood (REML) and Hartung-Knapp adjustment. We also performed subgroup analyses based on age, sex, presence of a shockable initial cardiac rhythm, history of coronary artery disease, presence of an ischemic event as the presumed cause of arrest, and time to return of spontaneous circulation (ROSC). We tested for treatment effect modification by subgroup using random effects models, applying the method of REML and Hartung-Knapp adjustment. We also conducted meta-regression analyses applying the method of REML according to baseline variables (including left-ventricular ejection fraction (LVEF), the proportion of patients with radial access for cardiac catheterization, the proportion of patients without clinically significant coronary artery disease, and the proportion of patients with three-vessel coronary artery disease) for death.

The Cochrane Q statistic and Higgins and Thompsons’ I^2^ were calculated to assess study heterogeneity. An I^2^ of less than 25% was considered to indicate low heterogeneity, 25% to 75% was considered to be moderate heterogeneity, and above 75% was considered to be high heterogeneity [19]. Publication-bias and small-study bias were assessed using funnel plots and Egger’s regression test. All reported P values are two-sided, and no adjustments for multiple testing were performed. All statistical analyses were performed using R version 4.2.0 (R Core Team, Vienna, Austria) and the R package metafor (version 3.4.0).

## Results

### Study population

**Fig. 1:**
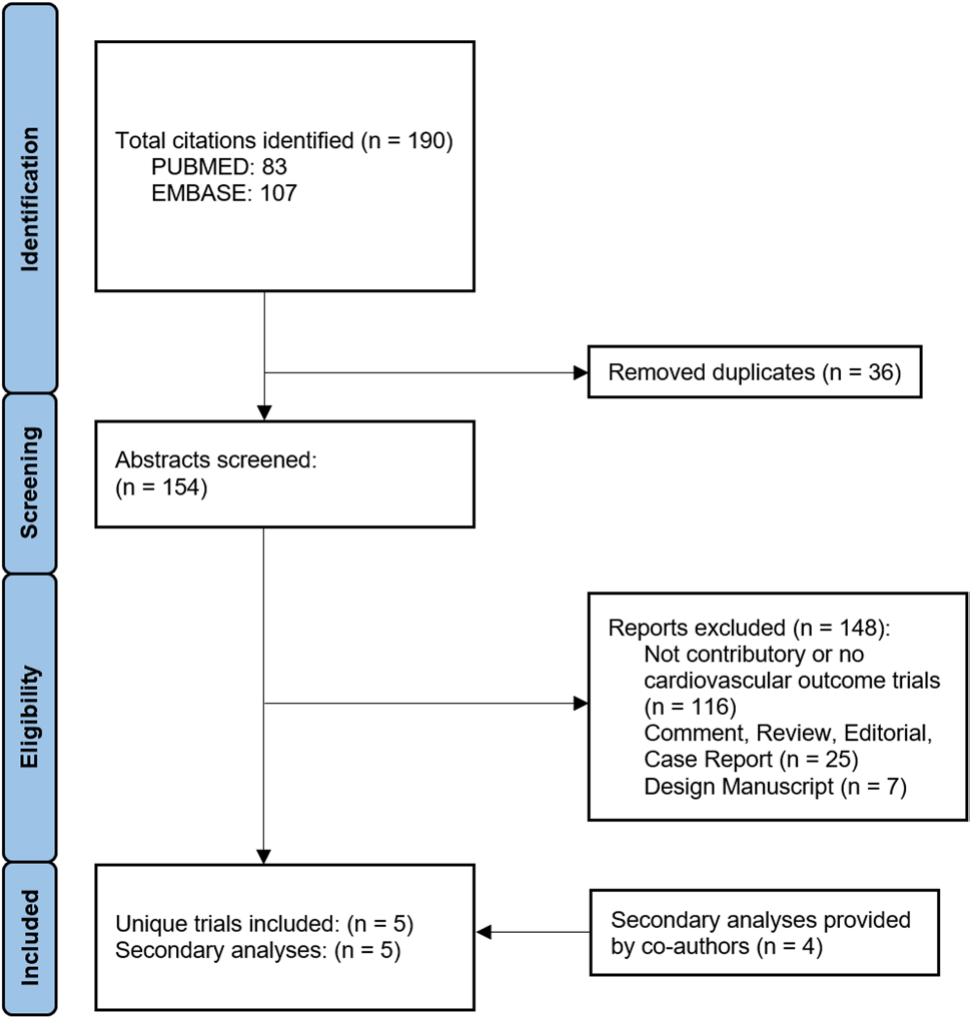
Prisma flow diagram summarizing the search strategy and selection of trials

In total, 1512 patients from 5 randomized controlled trials were included in the present meta-analysis (Fig. 1). All trials were well conducted and had a low risk of bias (Fig. S1). Funnel plots showed no significant publication bias (Fig. S2). The follow-up time of the primary trials ranged from 30 to 180 days. The weighted mean age was 67 years, and 394 (26%) patients were female (Table 1). In total, 255 (23%) had a prior myocardial infarction, and 286 (24%) had diabetes. The proportion of patients with an initial shockable rhythm ranged from 30% in the EMERGE trial to 100% in the COACT trial.

**Table 1:** Summary of the included trials

Characteristics	COACT [10]	PEARL [7]	TOMAHAWK [1]	COUPE [9]	EMERGE [8]	Total
Trial participants	538	99	530	66	279	1512
Follow-up (days)	90*	180	30	180	180	95
Age (years)	65 (48–82)	65 (57–73)	70 (60–78)	63 (56–72)	67 (55–76)	67
Female sex (%)	113 (21)	21 (21)	161 (30)	15 (23)	84 (30)	394 (26.1)
Smoking (%)	117/498 (23.5)	NA	108/335 (32.2)	17/66 (25.8)	NA	242/899 (26.9)
Hypertension (%)	257/534 (48.1)	55/99 (55.6)	323/474 (68.1)	44/66 (66.7)	NA	679/1173 (57.9)
Diabetes (%)	99/537 (18.4)	27/99 (27.3)	145/495 (29.3)	15/66 (22.7)	NA	286/1197 (23.9)
PAD (%)	39/537 (7.3)	6/99 (6.1)	48/444 (10.8)	8/66 (12.1)	NA	101/1146 (8.8)
Coronary artery disease (%)	195/538 (36.2)	32/99 (32.3)	172/458 (37.6)	15/66 (22.7)	NA	414/1161 (35.7)
Prior myocardial infarction (%)	149/538 (27.7)	17/99 (17.2)	89/455 (19.6)	NA	NA	255/1092 (23.4)
Shockable rhythm (%)	538/538 (100.0)	75/99 (75.8)	268/483 (55.5)	54/66 (81.8)	88/271 (32.5)	1023/1457 (70.2)
Witnessed arrest (%)	421/538 (78.3)	90/99 (90.9)	462/516 (89.5)	NA	252/278 (90.6)	1225/1431 (85.6)
Bystander CPR (%)	NA	70/99 (70.7)	294/499 (58.9)	NA	191/247 (77.3)	555/845 (65.7)
Time to ROSC (minutes)	15	20	15	22	26	18
Targeted temperature management (%)	504/538 (93.7)	79/99 (80.0)	414/530 (78.1)	66/66 (100.0)	103/196 (52.6)	1166/1429 (81.6)
Time to CAG in the early/immediate treatment arm (hours)	2 (2–3)	2 (1–2)	3 (2–4)	3 (2–3)	2 (2–3)	2.4
Time to CAG in the delayed/selective treatment arm (hours)	122 (52–197)	60 (14–173)	47 (26–117)	129 (87–186)	66 (41–75)	81.6
PCI performed (%)	154/437 (35.2)	24/73 (32.9)	163/412 (39.6)	12/52 (23.1)	55/200 (27.5)	408/1174 (34.8)

Continuous variables are reported as medians (interquartile range) unless stated otherwise.

*CAG* coronary angiography, *CPR* cardiopulmonary resuscitation, *PAD* peripheral artery disease, *ROSC* return of spontaneous circulation, *PCI* percutaneous coronary intervention

*12 months data have been reported for death. Missing values have not been collected in the respective trials

### Early/immediate versus delayed/selective coronary angiography

The median time to coronary angiography ranged from 2 to 3 h (weighted mean 2.4 h) in patients assigned to early/immediate coronary angiography, compared to 47–129 h (weighted mean 81.6 h) in patients assigned to a strategy of delayed/selective coronary angiography. Coronary revascularization was performed in 246 (34%) patients treated with early/immediate coronary angiography and in 162 (36%) patients treated with delayed/selective coronary angiography.

**Fig. 2:**
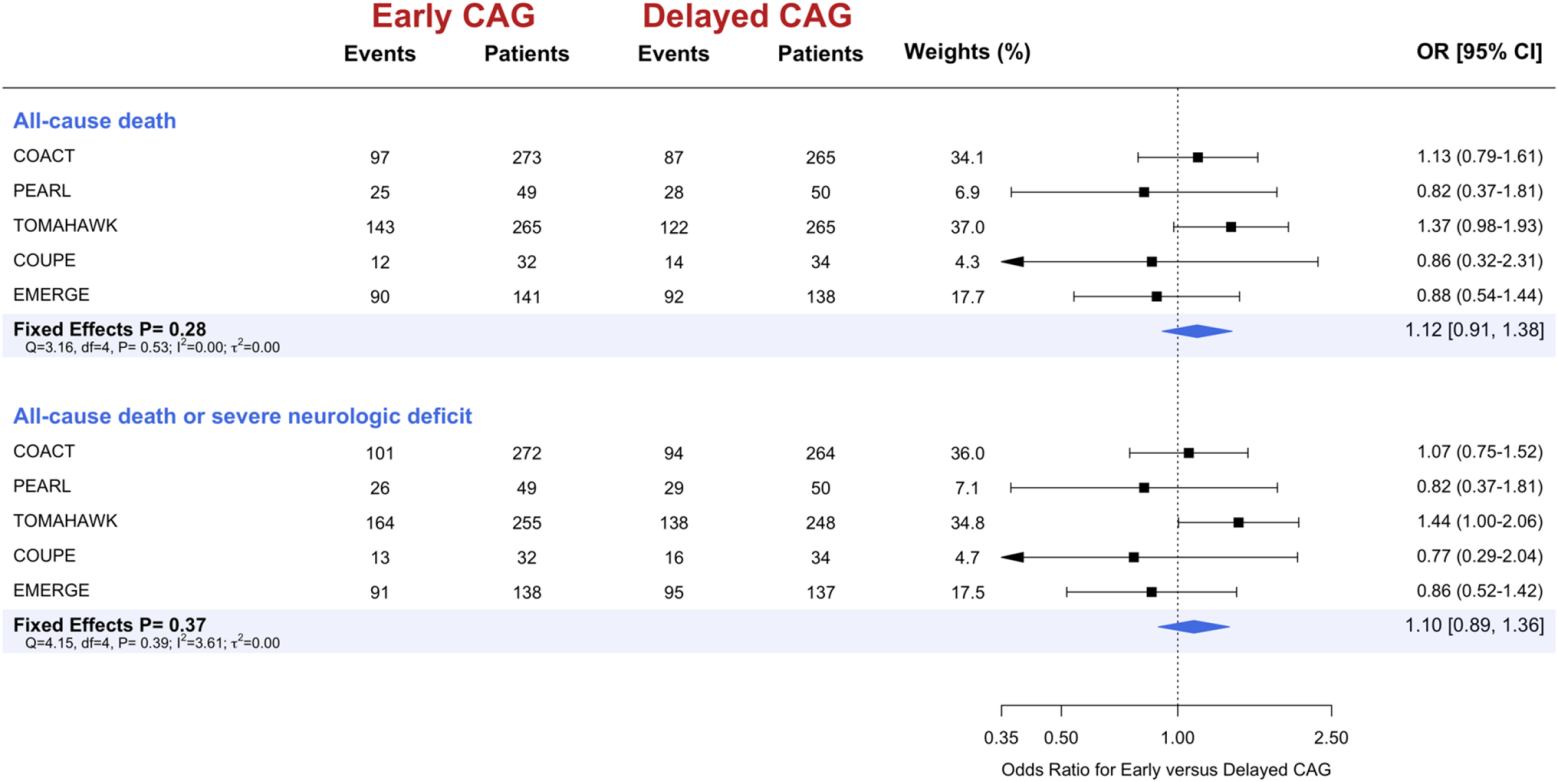
Pooled data for (**A**) all-cause death and (**B**) the composite outcome of all-cause death or neurological deficit. Legend: *CAG* coronary angiography; *OR* odds ratio; *CI* confidence interval

The primary outcome of all-cause death occurred in 367 patients of the 760 patients (48.3%) that were assigned to a strategy of early/immediate coronary angiography and in 343 patients of the 752 patients (45.6%) assigned to a strategy of delayed/selective coronary angiography, resulting in no significant difference in the odds of death (OR 1.12, 95% CI 0.91 to 1.38, P = 0.28, Fig. 2). The most common causes of death were anoxic brain injury (51.1%), followed by cardiovascular death (25.7%) and did not differ between the two treatment strategies (Supplemental Table 2). Baseline factors including LVEF, radial access for coronary angiography, the proportion of patients without significant coronary artery disease, and the presence of three-vessel coronary artery disease did not modify the effect of immediate/early versus delayed/selective coronary angiography on death in a meta-regression (P-interaction > 0.10 for each; Fig. S3).

Similarly, there was no significant difference in the odds of the composite of all-cause death or neurological deficit (OR 1.10, 95% CI 0.89 to 1.36, P = 0.37). Sensitivity analyses, that included the DISCO pilot study (Direct or Subacute Coronary Angiography in Out-of-Hospital Cardiac Arrest) [6] with a follow-up of 24 h only, or using random effects models yielded similar results (Figs. S4 and S5).

Moreover, there was no significant difference in safety events, including the odds of bleeding (OR 0.94, 95% CI 0.52 to 1.70, P = 0.83; Fig. S6) and kidney events (OR 1.13, 95% CI 0.75 to 1.70, P = 0.55; Fig. S7) between early/immediate versus delayed/selective coronary angiography.

### Subgroup analyses

**Fig. 3:**
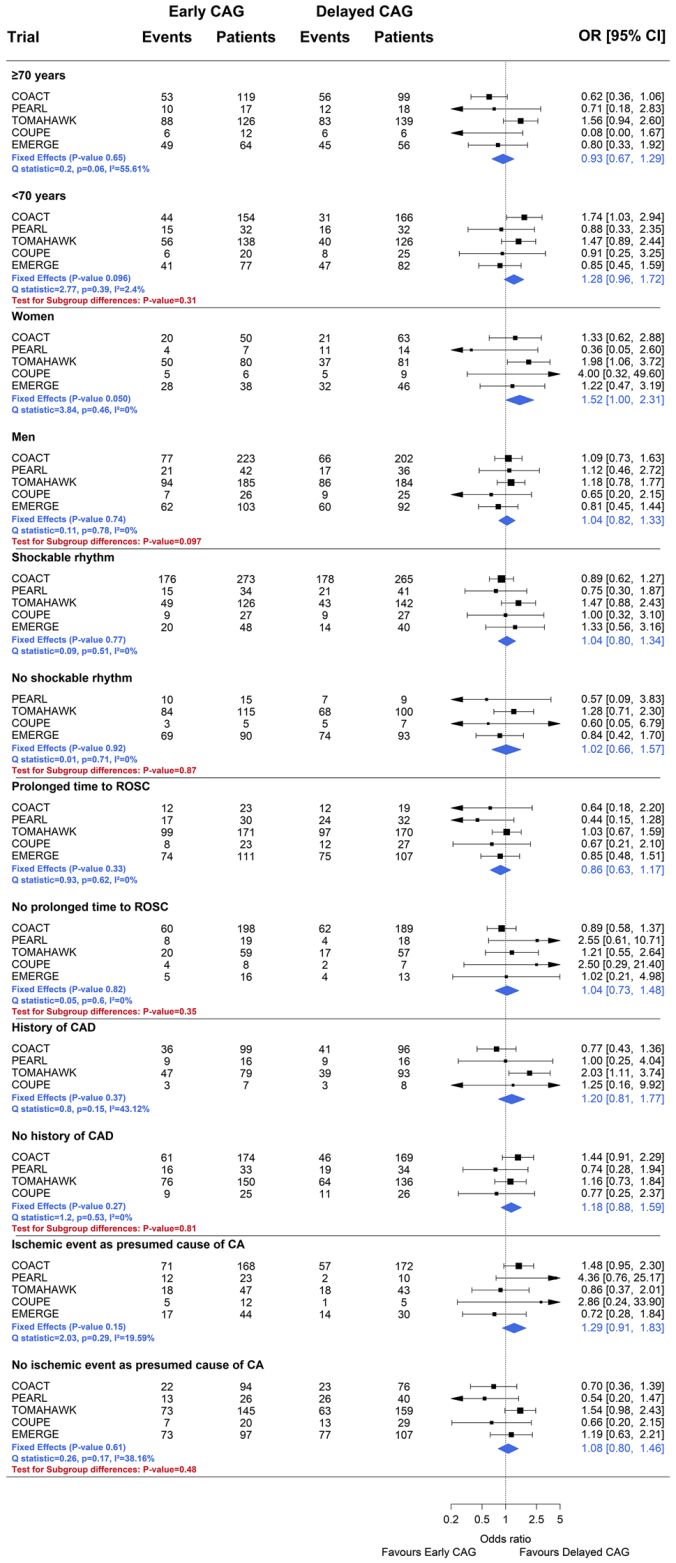
Pooled results of early versus delayed coronary angiography trials on all-cause death across subgroups. Prolonged time to ROSC was defined as > 15 min (except for COACT > 30 min). Legend: *CAG* coronary angiography; *OR* odds ratio; *CI* confidence interval

Early/immediate (versus delayed/selective) coronary angiography tended to be associated with higher odds of death in women (OR 1.52, 95% CI 1.00 to 2.31, P = 0.050) than in men (OR 1.04, 95% CI 0.82 to 1.33, P = 0.74; P-value for interaction 0.097; Fig. 3). A qualitatively similar relationship, though statistically not significant, was found in younger patients, where early/immediate coronary angiography was associated with higher odds of death (OR 1.28, 95% CI 0.96 to 1.72), which was not seen in older patients (OR 0.93, 95% CI 0.67 to 1.29, P-value for interaction 0.31). There was no evidence of effect modification for all-cause death across tested subgroups, including the presence or absence of an initial shockable cardiac rhythm, history of coronary artery disease, presence of an ischemic event as the presumed cause of the cardiac arrest, or time to ROSC (Fig. 3).

## Discussion

The present trial-level meta-analysis of 1512 patients with OHCA and no ST-segment elevation showed that a strategy of early/immediate versus delayed/selective coronary angiography did not reduce the risk of all-cause death. This finding was consistent across tested subgroups, including patients with and without an ischemic event as the presumed cause of the cardiac arrest. However, a strategy of early/immediate coronary angiography tended to be associated with increased odds of death in women as compared with men.

This meta-analysis included previously unreported data for subgroups and thus updates and expands on previous meta-analyses [20, 21]. The present findings corroborate that a strategy of early coronary angiography is not superior over a strategy of delayed/selective coronary angiography in OHCA patients without ST-segment elevation. Moreover, early coronary angiography may cause harm by increasing the odds of death in certain patient subgroups. There are several possibilities that may explain these findings. First, only a subset of patients (35%) required coronary revascularization and thus derived possible benefit from the performed procedure. These numbers imply that a non-selective “one size fits all” approach that refers all OHCA patients without ST-segment elevation to coronary angiography is not favorable. Future research should be directed at the early identification of patients with OHCA who may benefit from coronary revascularization. The majority of patients who did not require coronary revascularization was exposed to increased risk of peri- and postprocedural complications of coronary angiography, including bleeding events, stroke, or worsening of kidney function, although the complication rates were small across the included trials, and there was no significant difference between the treatment groups. Moreover, patients with OHCA frequently require hemodynamic monitoring, ventilation, and early initiation of targeted temperature management. An early invasive coronary angiography approach may result in delay of essential quality critical care. In the COACT and TOMAHAWK trials, targeted temperature management was initiated on average approximately 30 to 60 min earlier in patients assigned to the delayed/selective strategy arm than in those randomized to the early strategy arm [1, 10]. The notion of a lack of benefit from immediate coronary angiography is also consistent with randomized controlled trials in non-ST-segment elevation myocardial infarction patients without cardiac arrest [22, 23].

The present study suggests that early coronary angiography is associated with higher odds of death in women than in men. This adds to the body of evidence of sex-based differences in outcomes after OHCA. Although women are less likely than men to develop coronary artery disease, and their severity of coronary artery disease is lower in OHCA [24, 25], they have a greater risk of major adverse cardiovascular events following percutaneous coronary intervention than men [15]. Importantly, women made up only one-fourth of the trial population. It is therefore crucial to increase the proportion of women participating in clinical trials to ensure equal representation.

In the present meta-analysis, comprising more than 1,500 patients across 5 randomized trials among patients with OHCA and no ST-elevation on the initial ECG, we were unable to identify any subgroup of patients, based on demographics or clinical presentation, which derived potential benefit from an early invasive strategy. Whether specific subgroups of patients not explored here may derive potential benefit remains unclear. The ongoing DISCO (Direct or Subacute Coronary Angiography in Out-of-hospital Cardiac Arrest; ClinicalTrials.gov Identifier: NCT02309151) trial will provide further insight into sex differences and potential identification of other high-risk subgroups.

## Limitations

Several limitations of this meta-analysis need to be acknowledged. This was a trial level meta-analysis of five randomized controlled trials rather than individual participant study level data. The definitions and time points of immediate/early and delayed/selective coronary angiography as well as the follow-up varied between trials. Moreover, we were not able to investigate additional subgroups of interest, such as those stratified by baseline LVEF, interventional access site, severity of coronary artery disease, or cardiac biomarker concentrations. Furthermore, the study is limited by the absence of information regarding timing and use of dual antiplatelet therapy regimens, guideline directed medical therapy, and medications at discharge. In addition, baseline characteristics, mode of death, and endpoints beyond all-cause death have not been reported for subgroups. Although all trials included in the present meta-analysis were well conducted, treating clinicians were aware of treatment assignment by the inherent nature of the trial design. Moreover, cross-over rates ranged between 14 and 31% across the trials and the follow up times differed. Lastly, all trials excluded patients with ST-segment elevation or in-hospital cardiac arrest, and only a limited number of patients with hemodynamic or electrical instability were included.

## Conclusions

In conclusion, there was no statistically significant difference between immediate/early and delayed/selective coronary angiography in OHCA patients without ST-segment elevation with regard to all-cause mortality or neurologic impairment. We did not identify any subgroup of patients, based on demographics or clinical presentation, which derived potential benefit from an early invasive strategy. Though, women tended to have higher odds of death with early/immediate coronary angiography than men.

### Electronic supplementary material

Below is the link to the electronic supplementary material.

Supplementary file1 (DOCX 428 KB)

## Data Availability

The authors declare that all supporting data are available within the article and its online-only Data Supplement.

## Appendix

### Search algorithms

#### Pubmed search

(“Out-of-Hospital Cardiac Arrest” [Mesh] OR “OHCA” [tiab] OR “Out-of-Hospital Cardiac arrest” [tiab] OR “heart arrest” [tiab]) AND (“coronary angiography” [Mesh] OR “coronary angiogram” [tiab] OR “PCI” [tiab] OR “CAG” [tiab]) AND (random*[tw] OR “Letter”[pt] OR “trial” [tiab]) NOT (“Review” [ptyp]).

#### Embase search

(‘out of hospital cardiac arrest’/exp OR ‘out of hospital cardiac arrest’:ti,ab OR ‘OHCA’:ti,ab) AND (‘coronary angiography’/exp OR ‘coronary angiography’:ti,ab OR ‘PCI’:ti,ab OR ‘coronary angiogram’:ti,ab OR ‘percutaneous coronary intervention’:ti,ab) AND (‘trial’:ti,ab) NOT (‘chapter’/it OR ‘conference review’/it OR ‘editorial’/it OR ‘review’/it OR ‘meta-analysis’:ti).

## References

[1] Desch S, Freund A, Akin I (2021). Angiography after out-of-hospital cardiac arrest without ST-segment elevation. N Engl J Med.

[2] Kleissner M, Sramko M, Kohoutek J, Kautzner J, Kettner J (2015). Impact of urgent coronary angiography on mid-term clinical outcome of comatose out-of-hospital cardiac arrest survivors presenting without ST-segment elevation. Resuscitation.

[3] Roffi M, Patrono C, Collet J-P (2016). 2015 ESC Guidelines for the management of acute coronary syndromes in patients presenting without persistent ST-segment elevation. Eur Heart J.

[4] Myat A, Song KJ, Rea T (2018). Out-of-hospital cardiac arrest: current concepts. Lancet.

[5] Samsky MD, Morrow DA, Proudfoot AG (2021). Cardiogenic shock after acute myocardial infarction: a review. JAMA.

[6] Elfwén L, Lagedal R, Nordberg P (2019). Direct or subacute coronary angiography in out-of-hospital cardiac arrest (DISCO)—an initial pilot-study of a randomized clinical trial. Resuscitation.

[7] Kern KB, Radsel P, Jentzer JC (2020). Randomized pilot clinical trial of early coronary angiography versus no early coronary angiography after cardiac arrest without ST-segment elevation: the PEARL study. Circulation.

[8] Hauw-Berlemont C, Lamhaut L, Diehl JL (2022). Emergency vs delayed coronary angiogram in survivors of out-of-hospital cardiac arrest: results of the randomized. Multicentric EMERGE Trial JAMA Cardiol.

[9] Viana-Tejedor A, Andrea-Riba R, Scardino C (2022). Coronary angiography in patients without ST-segment elevation following out-of-hospital cardiac arrest. Rev Esp Cardiol (Engl Ed).

[10] Lemkes JS, Janssens GN, van der Hoeven NW (2019). Coronary angiography after cardiac arrest without ST-segment elevation. N Engl J Med.

[11] Safdar B, Stolz U, Stiell IG (2014). Differential survival for men and women from out-of-hospital cardiac arrest varies by age: results from the OPALS study. Acad Emerg Med.

[12] Morrison LJ, Schmicker RH, Weisfeldt ML (2016). Effect of gender on outcome of out of hospital cardiac arrest in the resuscitation outcomes consortium. Resuscitation.

[13] Winther-Jensen M, Hassager C, Kjaergaard J (2018). Women have a worse prognosis and undergo fewer coronary angiographies after out-of-hospital cardiac arrest than men. Eur Heart J Acute Cardiovasc Care.

[14] Blom MT, Oving I, Berdowski J (2019). Women have lower chances than men to be resuscitated and survive out-of-hospital cardiac arrest. Eur Heart J.

[15] Kosmidou I, Leon MB, Zhang Y (2020). Long-term outcomes in women and men following percutaneous coronary intervention. J Am Coll Cardiol.

[16] Mody P, Pandey A, Slutsky AS (2021). Gender-based differences in outcomes among resuscitated patients with out-of-hospital cardiac arrest. Circulation.

[17] Shamseer L, Moher D, Clarke M (2015). Preferred reporting items for systematic review and meta-analysis protocols (PRISMA-P) 2015: elaboration and explanation. Bmj.

[18] Sterne JAC, Savović J, Page MJ (2019). RoB 2: a revised tool for assessing risk of bias in randomised trials. Bmj.

[19] Higgins JP, Thompson SG, Deeks JJ, Altman DG (2003). Measuring inconsistency in meta-analyses. BMJ.

[20] Verma BR, Sharma V, Shekhar S (2020). Coronary angiography in patients with out-of-hospital cardiac arrest without ST-segment elevation: a systematic review and meta-analysis. JACC Cardiovasc Interv.

[21] Freund A, van Royen N, Kern KB (2022). Early coronary angiography in patients after out-of-hospital cardiac arrest without ST-segment elevation: meta-analysis of randomized controlled trials. Catheter Cardiovasc Interv.

[22] Jobs A, Mehta SR, Montalescot G (2017). Optimal timing of an invasive strategy in patients with non-ST-elevation acute coronary syndrome: a meta-analysis of randomised trials. Lancet.

[23] Barbarawi M, Zayed Y, Kheiri B (2019). Optimal timing of coronary intervention in patients resuscitated from cardiac arrest without ST-segment elevation myocardial infarction (NSTEMI): a systematic review and meta-analysis. Resuscitation.

[24] Lindgren E, Covaciu L, Smekal D (2019). Gender differences in utilization of coronary angiography and angiographic findings after out-of-hospital cardiac arrest: a registry study. Resuscitation.

[25] Spoormans EM, Lemkes JS, Janssens GN (2021). Sex differences in patients with out-of-hospital cardiac arrest without ST-segment elevation: a COACT trial substudy. Resuscitation.

