# Supplementary material for: Early versus delayed coronary angiography in patients with out-of-hospital cardiac arrest and no ST-segment elevation: a systematic review and meta-analysis of randomized controlled trials
Source: Clin Res Cardiol. 2023 Jul 27;113(4):561–9. doi: 10.1007/s00392-023-02264-7 (PMC10954865; doi:10.1007/s00392-023-02264-7)

# Supplemental Material

Search algorithms

**Pubmed Search**

(“Out-of-Hospital Cardiac Arrest” [Mesh] OR “OHCA” [tiab] OR “Out-of-Hospital Cardiac arrest” [tiab] OR “heart arrest” [tiab]) AND (“coronary angiography” [Mesh] OR “coronary angiogram” [tiab] OR “PCI” [tiab] OR “CAG” [tiab]) AND (random*[tw] OR "Letter"[pt] OR “trial” [tiab]) NOT (“Review” [ptyp])

**Embase Search**

('out of hospital cardiac arrest'/exp OR 'out of hospital cardiac arrest':ti,ab OR 'OHCA':ti,ab) AND ('coronary angiography'/exp OR 'coronary angiography':ti,ab OR ‘PCI':ti,ab OR ‘coronary angiogram’:ti,ab OR ‘percutaneous coronary intervention’:ti,ab) AND ('trial':ti,ab) NOT ('chapter'/it OR 'conference review'/it OR 'editorial'/it OR 'review'/it OR 'meta-analysis':ti)

### Supplemental Table 1: Definitions of interventions and outcomes across trials

| **Trial** | **COACT^26^** | **PEARL^7^** | **TOMAHAWK^1^** | **COUPE^9^** | **EMERGE^8^** |
| --- | --- | --- | --- | --- | --- |
| **Intervention** | CAG within 2 hours versus CAG after neurological recovery (Generally after discharge from intensive care unit) | CAG (<120 minutes) versus No CAG within 6 hours of admission | Immediate CAG versus delayed/selective CAG (>24 hours) | CAG within 2 hours versus CAG after neurological recovery (Generally before discharge from intensive care unit) | Emergent CAG versus delayed CAG (48-96 hours) |
| **Eligibility criteria** | Comatose OHCA patients with initial shockable rhythm and without ST-segment elevation or new LBBB, or prolonged refractory hemodynamic instability | Comatose OHCA patients  of presumed cardiac etiology without ST-segment elevation or new LBBB. | OHCA patients with no ST-segment  elevation or LBBB and no hemodynamic- or electrical instability. | Comatose OHCA patients with no ST-segment elevation or new LBBB. | OHCA patients with no ST-segment elevation. |
| **All-cause mortality** | All-cause death at 90 days. (Follow-up data at 365 days reported). | All-cause death at 180 days | All-cause death at 30 days | All-cause death at 30 days | All-cause death at 180 days |
| **Neurological deficit** | CPC-score 3-5 at 90 days | CPC-score 3-5 or modified Rankin score ≥4 at 30 days | CPC-score 3-5 at 30 days | CPC-score 3-5 at discharge | CPC-score 3-5 at 180 days |
| **All-cause mortality or neurological deficit** | All-cause mortality and or severe neurological deficit (CPC score 3-5) at 90 days | Reciprocal of intact functional status at discharge (CPC ≤2) | All-cause mortality and or severe neurological deficit (CPC score 3-5) at 30 days | Reciprocal of survival with good neurological outcome (CPC 1-2) | All-cause mortality and or severe neurological deficit (CPC score 3-5) at 180 days |
| **Bleeding** | Major TIMI bleeding | Bleeding requiring transfusion or intervention (BARC 2-5) | Moderate and severe bleeding (BARC 2-5) | Moderate and severe bleeding (BARC 2-5) | NA |
| **Acute renal worsening** | Need for renal replacement therapy | Acute renal dysfunction (>0.5 dL/mL increase in creatinine from baseline) | Acute renal failure requiring renal replacement therapy | Increase in creatinine > 0.5 mg/dL or > 50% baseline | NA |

Legend: BARC=Bleeding Academic Research Consortium; CAG=Coronary angiography; CPC= Cerebral performance category; LBB= Left bundle branch block; OHCA= Out-of-hospital cardiac arrest; ROSC=Return of spontaneous circulation; TIMI=Thrombolysis in Myocardial Infarction

### Supplemental Table 2: Causes of death stratified by treatment strategy

|  | COACT | | PEARL | | TOMAHAWK | | COUPE | | EMERGE | |
| --- | --- | --- | --- | --- | --- | --- | --- | --- | --- | --- |
| Treatment arm | **Early/**  **Immediate CAG** | **Delayed/**  **Selective CAG** | **Early/**  **Immediate CAG** | **Delayed/**  **Selective CAG** | **Early/**  **Immediate CAG** | **Delayed/**  **Selective CAG** | **Early/**  **Immediate CAG** | **Delayed/**  **Selective CAG** | **Early/**  **Immediate CAG** | **Delayed/**  **Selective CAG** |
| All-cause death | 94 | 83 | 24 | 29 | 143* | 122* | 12 | 14 | 90 | 92 |
| Cardiovascular death | 18 (19%) | 12 (14%) | 6 (25%) | 6 (21%) | 49 (35%) | 35 (29%) | - | - | - | - |
| Sudden cardiac death | - | - | - | - | 6 (4%) | 0 (0%) | - | - | - | - |
| Cardiogenic shock | 11 (12%) | 7 (8%) | - | - | 35 (25%) | 28 (24%) | - | - | - | - |
| Hemorrhagic shock | - | - | - | - | 1 (1%) | 0 (0%) | - | - | - | - |
| Arrhythmia | 7 (7%) | 5 (6%) | - | - | - | - | - | - | - | - |
| Stroke | - | - | - | - | 3 (2%) | 2 (2%) | - | - | - | - |
| Neurological injury/  anoxic brain injury | 59 (63%) | 55 (66%) | 16 (67%) | 17 (59%) | 52 (38%) | 49 (42%) | - | - | - | - |
| Multi organ failure/sepsis | 8 (9%) | 12 (14%) | - | - | 10 (7%) | 16 (14%) | - | - | - | - |
| Other causes | 9 (10%) | 4 (5%) | 2 (8%) | 6 (21%) | 31 (23%) | 22 (19%) | - | - | - | - |

*Information on the primary cause of death was available in 255 of 265 deceased patients.

### Supplemental Figure 1: Risk of bias assessment

| **Unique Trial** | **D1** | **D2** | **D3** | **D4** | **D5** | **Overall** |  |  |  |
| --- | --- | --- | --- | --- | --- | --- | --- | --- | --- |
| COACT |  |  |  |  |  |  |  |  |  |
| PEARL |  |  |  |  |  |  |  |  | Low risk |
| TOMAHAWK |  |  |  |  |  |  |  |  | Some concerns |
| COUPE |  |  |  |  |  |  |  |  | High risk |
| EMERGE |  |  |  |  |  |  |  |  |  |

Legend: D1 = Randomization process; D2= Deviations from the intended interventions; D3= Missing outcome data; D4= Measurement of the outcome; D5= Selection of the reported result

### Supplemental Figure 2: Funnel plot for all-cause death


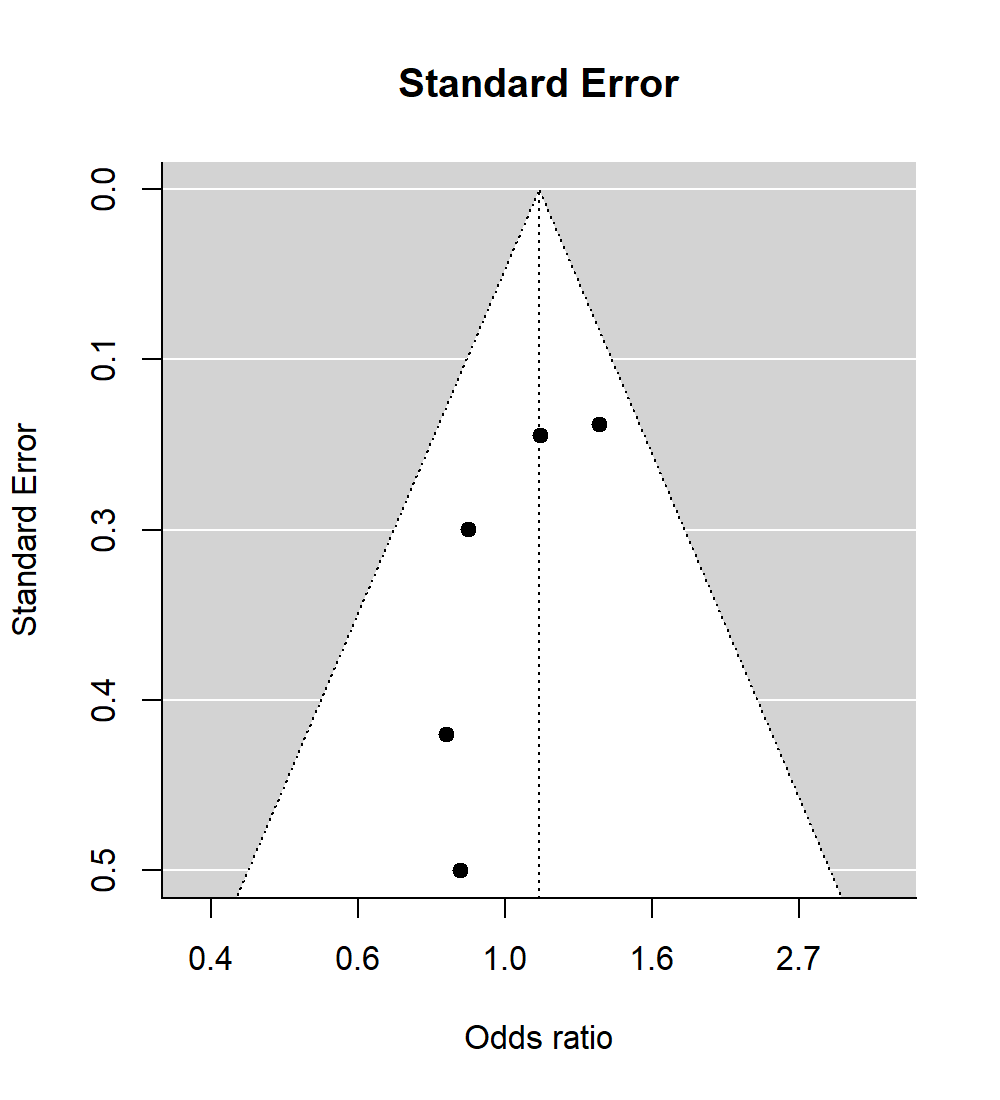


Supplemental Figure 3: Meta-regression of the relationship between all-cause death and A.) LVEF, B.) proportion of patients with radial access for coronary catheterization, C.) without clinically significant coronary artery disease, and D.) with three-vessel coronary artery disease (D).

LVEF was assessed upon admission in all trials except for the COACT trial, which measured it with a median delay of five days.

Legend: LVEF= Left-ventricular ejection fraction; CAD=Coronary artery disease


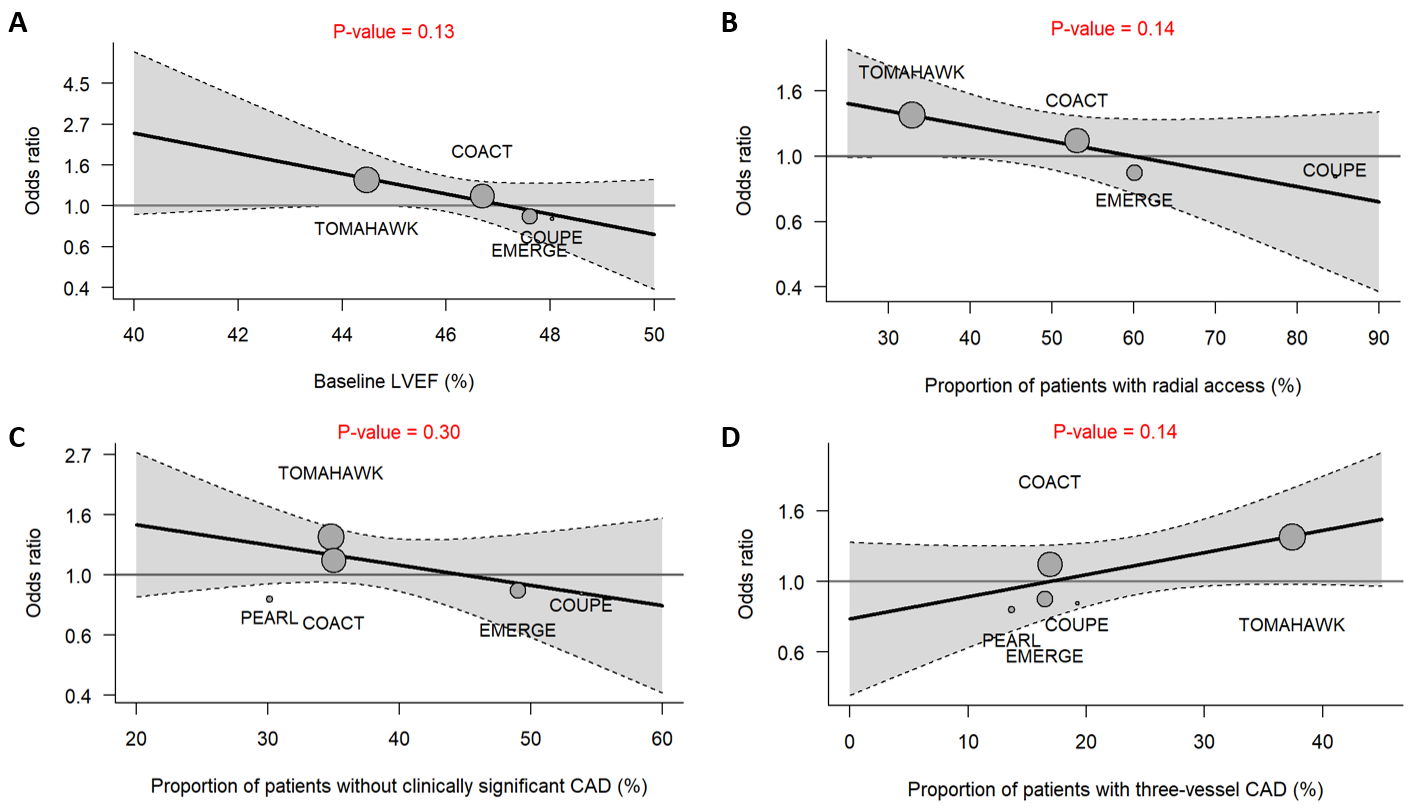


Supplemental Figure 4: Meta-analysis of early versus delayed coronary angiography using fixed-effects on all-cause mortality including randomized controlled trials with a follow-up duration under 30 days.

There was no evidence of heterogeneity between trials (Q statistic=0.89, P=0.49, I²=0%).

Legend: CAG = coronary angiography; OR = odds ratio; CI = confidence interval.


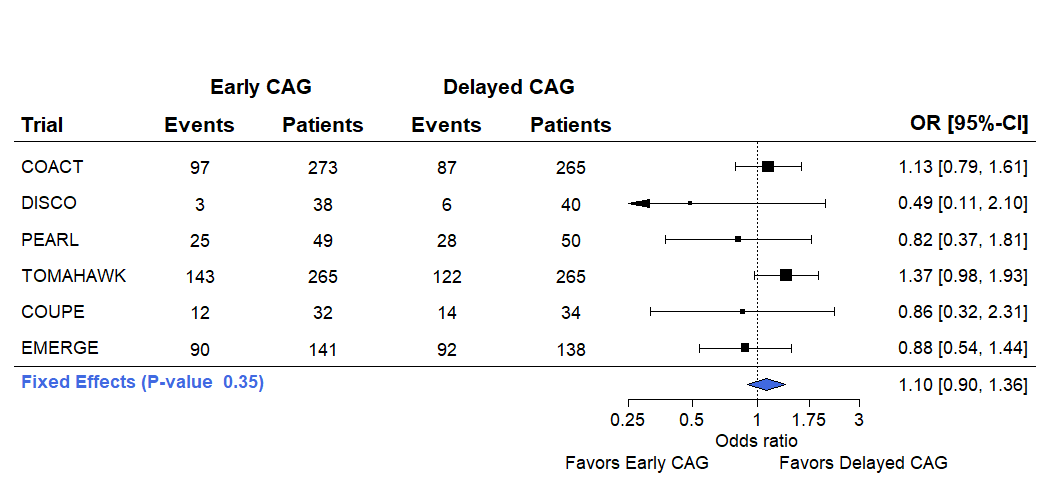


### Supplemental Figure 5: Meta-analysis of early versus delayed coronary angiography using *random-effects* on (A) all-cause mortality and (B) the composite outcome of all-cause mortality or neurological deficit.

Legend: CAG = coronary angiography; OR = odds ratio; CI = confidence interval.

**A**

**
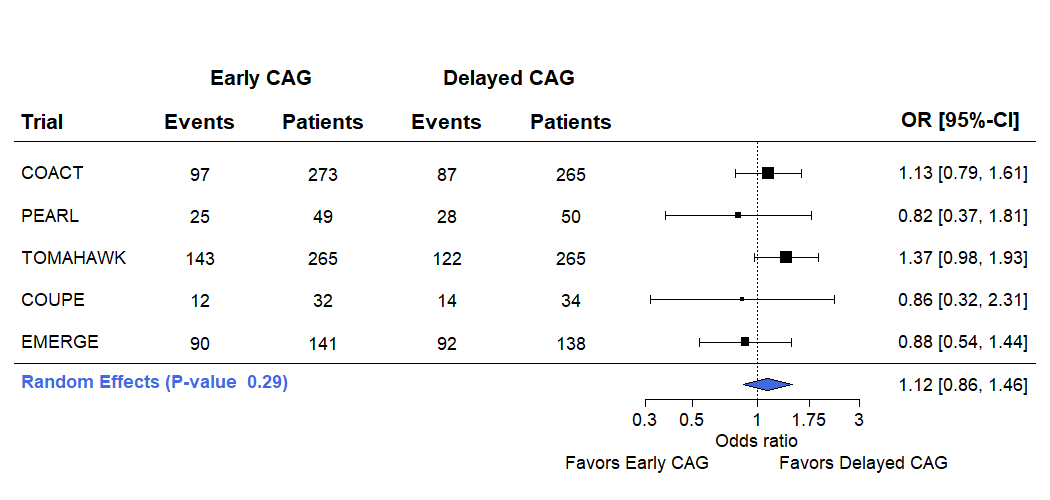
**

**B**


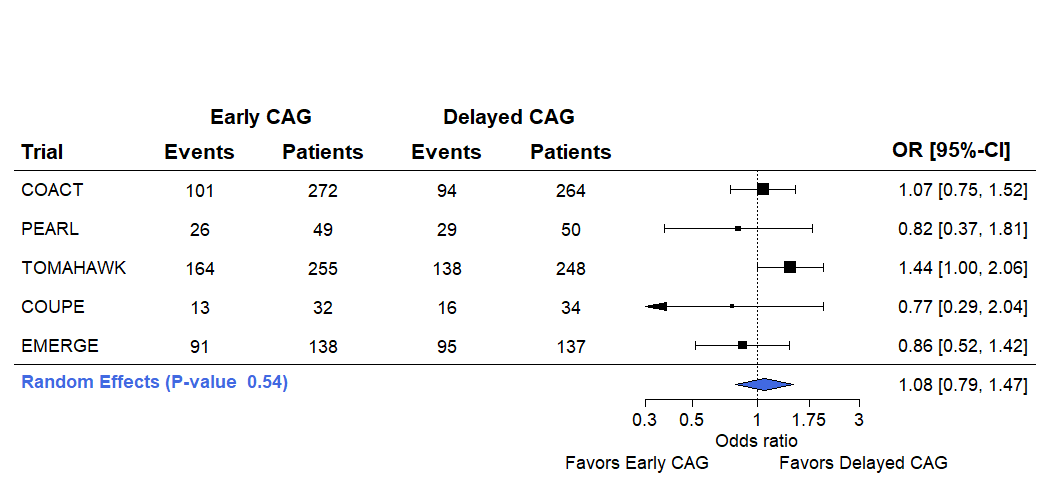


Supplemental Figure 6: Meta-analysis of early versus delayed coronary angiography on bleeding events.

There was no significant heterogeneity between trials: Q statistic=0.05, p=0.33, I²=12.53%.

Legend: CAG = coronary angiography; OR = odds ratio; CI = confidence interval.


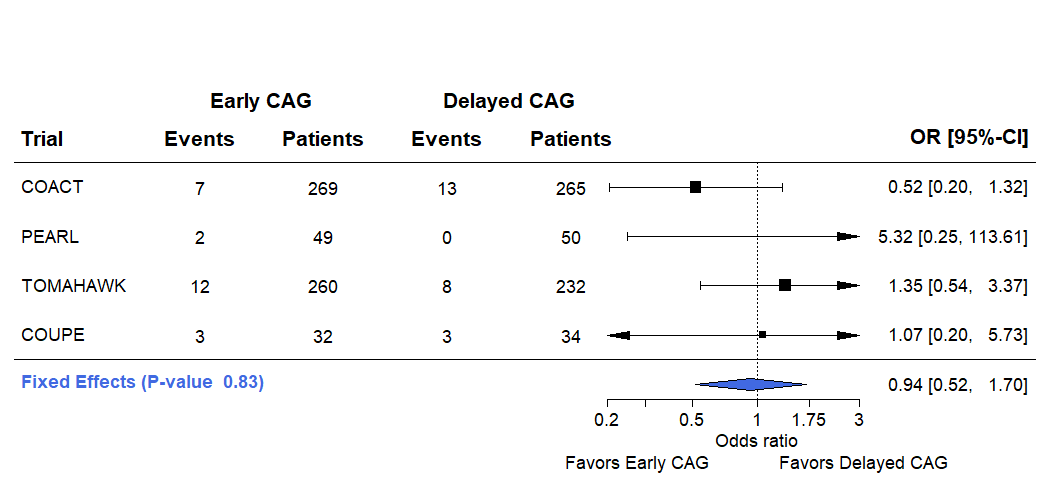


Supplemental Figure 7: Meta-analysis of early versus delayed coronary angiography on worsening of kidney function.

There was moderate heterogeneity between trials: Q statistic=0.36, p=0.21, I²=33.84%.

Legend: CAG = coronary angiography; OR = odds ratio; CI = confidence interval.


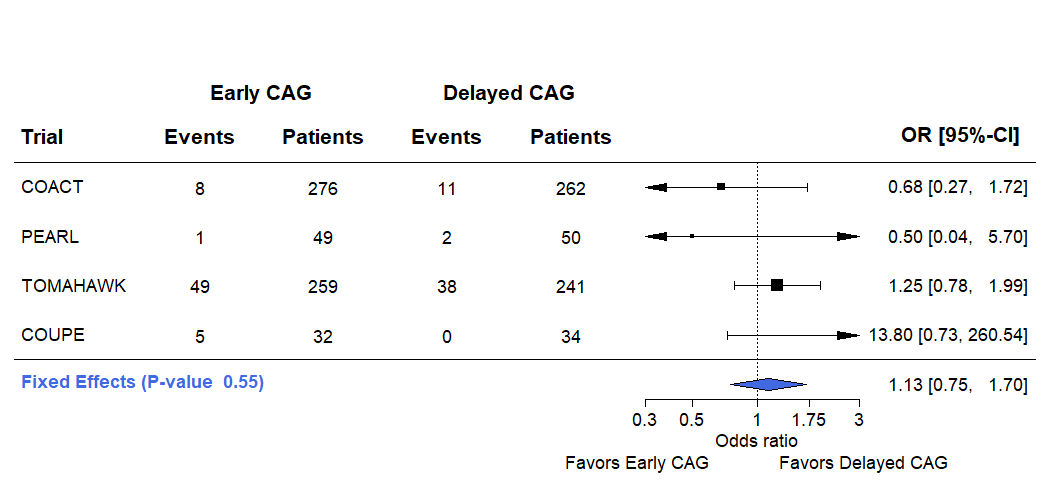

Supplement: Supplementary file 1 — Supplementary file1 (DOCX 428 KB) [file 392_2023_2264_MOESM1_ESM.docx]
